# Activation of the Oxytocin System in the Hypothalamic Paraventricular Nucleus Improves Stress-Induced Postpartum Depression-Like Behavior in Rats

**DOI:** 10.62641/aep.v53i3.1773

**Published:** 2025-05-05

**Authors:** Jingjing Dang, Huihui Kuai, Siqi Zhou, Shanshan Guo, Jingyi Sheng, Zhiping Wang

**Affiliations:** ^1^Nanjing Medical University, 210000 Nanjing, Jiangsu, China; ^2^Department of Anesthesiology, The Affiliated Hospital of Xuzhou Medical University, 221000 Xuzhou, Jiangsu, China; ^3^Jiangsu Province Key Laboratory of Anesthesiology, Xuzhou Medical University, 221000 Xuzhou, Jiangsu, China; ^4^Department of Gastroenterology, Nanjing Drum Tower Hospital Clinical College of Jiangsu University, 210000 Nanjing, Jiangsu, China

**Keywords:** postpartum depression, oxytocin, paraventricular nucleus, chemogenetic activation

## Abstract

**Background:**

Oxytocin (OT) is a key molecule that not only acts as a uterine-contracting hormone during delivery but is also a critical maternal hormone that enables the social transmission of maternal behavior. Postpartum depression (PPD) is a series of depression-like symptoms that occur especially in women in the perinatal period and is accompanied by the failure to adapt to motherhood as well as impaired parent-infant bonding. However, the mechanism by which OT regulates PPD is still unclear. This study aimed to investigate the correlation between OT levels in the paraventricular nucleus (PVN) and PPD and to explore the potential mechanism underlying the involvement of the OT system in the regulation of PPD.

**Methods:**

We induced perinatal chronic stress in pregnant rats to establish a PPD model. OT levels in the cerebrospinal fluid (CSF) and PVN were measured throughout the perinatal period. We administered the chemogenetic virus hM3Dq into the PVN, intraperitoneally injected N-oxyclozapine to activate OT-secreting neurons, and observed the effects of OT treatment on behaviors related to PPD. Finally, we investigated the potential mechanism underlying PPD regulation by the OT system via transmission electron microscopy, immunofluorescence (IF), and quantitative real-time PCR (qRT-PCR).

**Results:**

Compared with those in the normal group, CSF oxytocin levels in the postpartum depression group decreased from late pregnancy to lactation (*p* < 0.001). Chemogenetic activation-induced endogenous OT release in the PVN not only alleviated PPD-like symptoms in rats but also enhanced the intracellular production of OT. Transmission electron microscopy revealed an increase in the size of the Golgi apparatus, endoplasmic reticulum, and dense vesicles within OT neurons. IF and qRT-PCR revealed elevated OT levels and increased oxytocin expression within the PVN following chemogenetic activation (*p* < 0.01).

**Conclusion:**

Lower OT levels are strongly associated with the occurrence of PPD. The release of activated OT has been shown to improve PPD-like behaviors in rats and promote intracellular OT synthesis.

## Introduction

Postpartum depression (PPD) is defined by the DSM-5 (Diagnostic and Statistical Manual of Mental Disorders-5th Edition) as a major depressive episode with onset beginning during pregnancy or within the first four weeks postpartum [1]. PPD affects 17.22% of the world’s population [2], indicating that the perinatal stage is a vulnerable period for the development of depression. The physiopathology of PPD involves multiple mechanisms [3], and hormonal changes during the perinatal period may amplify the influence of other risk factors and play a central role.

Oxytocin (OT) is a polypeptide consisting of nine amino acids. It is secreted by the cells of the supraoptic nucleus and paraventricular nucleus (PVN) of the hypothalamus. Studies involving rodents have implicated the role of OT in social behavior, social recognition, and various emotional responses [4, 5, 6]. However, the efficacy of oxytocin in treating women with PPD remains controversial [7], which may be related to the unclear relationship between OT and PPD.

Plasma OT levels fluctuate under the influence of estrogen during pregnancy and the perinatal period and contribute to adaptation to motherhood [8] and parent–infant bonding [9]. In 2020, a systematic review revealed that eight of the 12 reviewed studies reported an inverse relationship between plasma OT levels and depressive symptoms [10]. However, some studies have reached different conclusions [11, 12], possibly because of the relatively independent OT systems between the peripheral and intracranial regions, and the concentration and changes in OT levels in the periphery cannot accurately reflect the dynamic changes in OT levels in the brain. Moreover, in a preliminary study, we found that the OT level in the cerebrospinal fluid (CSF) was more strongly correlated with the Edinburgh Postnatal Depression Scale (EPDS) at 3 months postpartum than those in the plasma or saliva and was more valuable in predicting PPD [13], which suggests that changes in CSF OT levels may reflect central OT release over an extended period.

Exposure to chronic stress during pregnancy is a major risk factor for PPD. Several studies have demonstrated that long-term chronic stress can change OT levels in the plasma through epigenetic mechanisms [9] or through interactions with the hypothalamic‒pituitary‒adrenal (HPA) axis [14], indirectly interfering with the OT system [15]. The exact mechanisms by which the OT system participates in stress-induced depression are still unknown.

With advancements in photogenetic and chemogenetic techniques, the activation of endogenous OT release in the hypothalamus has been investigated for its potential therapeutic effects in pain management [16], autism treatment [17], and fear-related disorders [18, 19]. In this study, we used chemogenetics to activate endogenous OT release and observed whether OT can alleviate PPD symptoms. We hypothesized that the OT level in CSF may reflect the OT level in the brain, that a lower OT level may predict a greater risk of PPD, and that increasing the endogenous OT level would be beneficial for improving postpartum depression-like symptoms.

## Materials and Methods

### Animals and PPD Model

Female Sprague-Dawley (SD) rats weighing 200–250 g (6–8 weeks, Xuzhou Medical University, Xuzhou, China) were individually housed with sexually experienced male rats for mating [20]. The presence of spermatozoa in the vaginal smear was used to mark Day 0.5 of gestation. At the initial stage of the experiment, the rats were randomly divided into two groups: (a) a naïve control group without induction of a depressive phenotype (Ctrl, n = 12) and (b) a group with PPD induced by chronic restraint stress [21] (PPD, n = 12). Throughout gestation, all the rats were left undisturbed. The PPD group underwent repeated episodes of restraint stress in a transparent cylinder measuring 7.5 cm in diameter and 15 cm in length under bright light for 2 h once daily (from 8:30 AM to 11:00 AM on Days 8–20 of gestation). Both groups of female rats experienced spontaneous labor and nursed their offspring.

### Hypothalamic PVN Virus Injection

In the second stage of the experiment, to manipulate OT neurons in the PVN, we used a designer receptor exclusively activated by designer drug (DREADD) approach [22]: adeno-associated viruses (AAVs) carrying rAAv-oxytocin CRE WPREs (PT-0263, AAV2/9, 5.10 × 10^12^ genomic copies/mL) combined with rAAV-Ef1a-DIO-*hM3D(Gq)*-EGFP-WPREs (PT-0816, AAV2/9, 5.92 × 10^12^ genomic copies/mL) or combined with rAAV-Ef1a-DIO-EGFP-WPREs (PT-0012, AAV2/9, 5.14 × 10^12^ genomic copies/mL) were obtained from Wuhan Shumi Brain Science and Technology Co., Ltd. (Wuhan, China). This Cre-Loxp-based dual-virus design achieved specific infection of OT-secreting cell genes, and the insertion of target gene fragments (*hM3Dq*-*eGFP* or *eGFP*) through DIO altered the activity of OT neurons via clozapine-N-oxide (CNO, 17366, MedChemExpress, Shanghai, China) administration.

For AAV injection, rats were anesthetized with sodium pentobarbital (40 mg/kg, i.p.) and placed on a stereotaxic instrument. With the help of a guide cannula, the double virus mixture was injected bilaterally into the PVN (coordinates: A/P, –0.85 mm posterior to bregma; M/L, 0.2 mm; D/V, –4.8 mm), and the syringe was not removed until 15–20 min after the end of infusion to allow time for the diffusion of the virus. After surgery, all the rats were returned to their home cages for one week for recovery.

The rats were then divided into four groups: two control groups (Ctrl/eGFP = 10 and Ctrl/hM3Dq = 10) and two PPD groups (PPD/eGFP = 10 and PPD/hM3Dq = 10). The experimental procedure is illustrated in Fig. 3. At the end of the experiment, the animals were put into a closed CO_2_ box and euthanized. All the animal experiments were conducted following the National Institutes of Health Guide for the Care and Use of Laboratory Animals and Care Committee of Xuzhou Medical University (approval number: 202208S018).

### Postpartum Behavioral Tests

#### Forced Swim Test (FST)

On the 5th postnatal day (PND5), the rats were subjected to the FST, and the entire process was conducted as previously described [23]. The instrument used was a cylindrical transparent vessel with the following parameters: 45 cm in height, 25 cm in diameter, 24 ± 1 °C in water temperature, and 35 cm in water depth. First, the rats were placed in the swimming pool and allowed 15 min of adaptive swimming training, after which they were removed, dried, and returned to their cages. After 24 h, the rats were placed in the cylindrical container for a 6 min test, and the cumulative immobility time was recorded during the last 5 min of the test. Immobility was defined as the state in which the rats floated in the water without struggling and only took the necessary actions to keep their head above the water. The assessment was performed by two independent researchers who were blinded to the experimental groups.

#### Open Field Test (OFT)

The OFT was performed on PND5-7 to analyze motor and exploratory behavior. The open field was made of black wood and consisted of a floor (60 × 60 cm) with 40 cm walls. The box floor was divided into 9 equal-sized squares. During a 10-min observation period, the rat was placed at one corner of the equipment facing the wall and allowed to explore freely. The total distance traveled, time spent in the central area, number of fecal pellets, number of line crossings and freezing time were recorded by an automated video-tracking system (ANY-maze, Stoelting Co., Chicago, IL, USA). The apparatus was thoroughly cleaned with 75% ethanol, followed by water between each test [24].

#### Sucrose Preference Test (SPT)

The SPT was conducted as previously described [25]. In the training phase, the rats were allowed to consume a 1% (w/v) sucrose solution from two bottles for 24 h. Then, the solution in one of the bottles was replaced with water for 24 h. In the detection phase, the rats were deprived of water and food for 24 h, and the SPT was then conducted starting at 9:00 a.m. Each rat was housed in an individual cage and given free access to two bottles containing 100 mL of sucrose solution (1%, w/v) and 100 mL of water. After 4 h, the consumed volumes (mL) of both the sucrose solution and water 8 were recorded. Sucrose preference was calculated as follows: sucrose preference (%) = sucrose consumption (mL)/(sucrose consumption [mL] + water consumption [mL]) × 100.

### Chemogenetic Activation of Oxytocin Oxy-Cre

After the induction of PPD, the rats were intraperitoneally injected with 0.5 mg/kg CNO every other day between 18:00 and 18:30 PM beginning on PND6. The administration of CNO combined with hM3Dq activated the OT-secreting cell membrane channels, promoting the release of endogenous OT. Rats exhibiting mistargeting of the surgical needle and expressing activated virus outside the PVN were excluded from the analysis. However, those with control virus expression outside the PVN were not excluded because their behavioral patterns were similar to those of the rats expressing enhanced green fluorescent protein (eGFP) in the correct location.

### Measurement of OT Levels in the CSF (ELISA)

Three days before pregnancy (P-3), 8 and 17 days after pregnancy (P8, P17), and 6 days postnatally (PND6), cerebrospinal fluid (CSF) was extracted. The OT concentration was measured via an oxytocin enzyme-linked immunosorbent assay (ELISA) Kit (Jiangsu Enzyme Exemption Industry Co., Ltd., Yancheng, China).

### Sample Collection and Histological Analysis

The rats were decapitated, and their brains were removed, fixed with 4% paraformaldehyde (158127, Sigma-Aldrich Trading Co., Ltd., Shanghai, China) at 4 °C overnight, and then transferred to 30% sucrose until they sank. To obtain the whole PVN, samples were sectioned from the optic chiasma on a vibratome (VT1000E; Leica Instruments, Heidelberg, BW, Germany) into 40 µm thick serial sections in the coronal plane, and a total of 50 sections were numbered and obtained.

### Immunofluorescence (IF)

Sections 20–25 were selected for staining. IF studies were performed to assess the number of OT cells and c-fos expression. Primary antibodies against OT (rabbit anti-OT, ab212193, Abcam, Shanghai, China) and c-fos (rabbit anti-c-fos, 2250, Cell Signaling Technology, Danvers, MA, USA) were diluted 1:500 in Tris-buffered saline (TBS). The fluorescence-tagged secondary antibody (goat anti-rabbit 594, ab150080, Abcam, Shanghai, China) was diluted 1:1000. Frozen brain sections were washed with phosphate-buffered saline (PBS) for 3 × 10 minutes and incubated with 10% goat serum for 2 hours. For the IF reaction, the sections were incubated overnight with the primary antibody, incubated for 2 h with the secondary antibody, washed with PBS for 3 × 10 min, mounted and sealed with DAPI (4^′^,6-Diamidino-2-phenylindole, C3362, APExBIO, Houston, TX, USA), naturally dried, and observed via fluorescence microscopy.

### IF Intensity Analysis

We performed semiquantitative analysis of the OT IF intensity via an Olympus laser confocal microscope and image analysis software (ImageJ, Version 1.52p, NIH, Bethesda, MD, USA). We manually circled the PVN region containing the OT-immunopositive neurons with a 10× objective lens, circled the “background” region without immunostaining near the OT-immunopositive region, and set the positive OT staining signal as an optical density (OD) greater than 2 times that of the background region. The image analysis software calculated the OT OD and the OT immunostaining positive signal coverage area (AreaMask). It multiplied the AreaMask of each film by the OD to obtain the OT integrated optical density (IOD) of the slice. The total IOD of OT immunoreactivity in the PVN was determined according to Cavalieri criteria.

### OT-Positive Cell Count

The sections with the largest coronal area of the PVN and sections immediately before and after the section with largest coronal area were selected for OT-positive cell counting. Finally, the number of OT cells from six slices from two rats in each group was included in the statistical analysis.

### IF Colabeling

The colocalization staining results were obtained via a multichromatographic imaging system (Olympus FV1000, Tokyo, Japan). We used image analysis software (FV10-ASW 4.2b, Tokyo, Japan) to separate and label the different dyes: the virus dye signal was labeled green, and the OT stain signal was labeled red. When the red and green markers overlapped, the virus and OT cells were considered colocalized.

### Transmission Electron Microscopy (TEM)

The PVN of the hypothalamic tissue from two female rats in each group were removed with a sharp blade to prepare tissue samples for electron microscopy. Within 1–3 min, the tissue samples were placed into an EP tube with fresh TEM fixative for further fixation and postfixation. The tissue was then dehydrated with ethanol, embedded, and moved to a 65 °C oven for polymerization for more than 48 h. The resin blocks were cut into 60–80 nm thick sections on an ultramicrotome (Leica EM UC7, Heidelberg, BW, Germany) and mounted onto 150 mesh cuprum grids for staining. Finally, the samples were observed and imaged via TEM (HT7800/HT7700, Hitachi, Tokyo, Japan).

### Quantitative Real-Time PCR (qRT-PCR)

Fresh hypothalamic PVN tissues were obtained from four female rats in each group.

Hypothalamic paraventricular nuclear RNA was extracted via the RNeasy Lipid Tissue Mini Kit (74804, Qiagen, China Co., Ltd., Shanghai, China), and the concentration of RNA was measured via a NanoDrop spectrophotometer (Thermo Scientific, Wilmington, DE, USA). Reverse transcription of 1 µg of RNA to cDNA was performed via a Transcriptor First Strand cDNA synthesis kit (4379012001, Roche, Indianapolis, IN, USA) according to the manufacturer’s instructions, and qRT-PCR was conducted as previously described [26] with the following primer sequences (see Table 1). The average of three technical replicates for each biological sample was normalized to glyceraldehyde 3-phosphate dehydrogenase (*GAPDH*) gene expression, and changes in gene expression levels were calculated via the 2^-Δ⁢Δ⁢Ct^ method.

**Table 1.** **Primers for PCR amplification**.

*OT*	F (CTGCGCTGCCAGGAGGAGAACT)
	R (AGCGCTCGGAGAAGGCAGACTC)
*GAPDH*	F (CAGTGCCAGCCTCGTCTCAT)
	R (AGGGGCCATCCACAGTCTTC)

*OT*, oxytocin; *GAPDH*, glyceraldehyde 3-phosphate dehydrogenase.

### Statistical Analysis

The Shapiro–Wilk test was used to test the normality of the distribution of the numerical data. Continuous variables with a normal distribution are expressed as the mean standard ± deviation (mean ± SD). Continuous variables that were not normally distributed are expressed as medians (interquartile ranges), and the Mann‒Whitney U test was used for the comparisons. Comparisons between two groups at a single time point were performed via *t* tests. Analysis of variance (ANOVA) was used for comparisons among the four groups (virus type × group). Statistical analyses were carried out in GraphPad Prism 5.0 (GraphPad Software, San Diego, CA, USA), and *p* < 0.05 was considered to indicate statistical significance.

## Results

### Depression-Like Behaviors are Induced by Chronic Restraint Stress

**Fig. 1.**
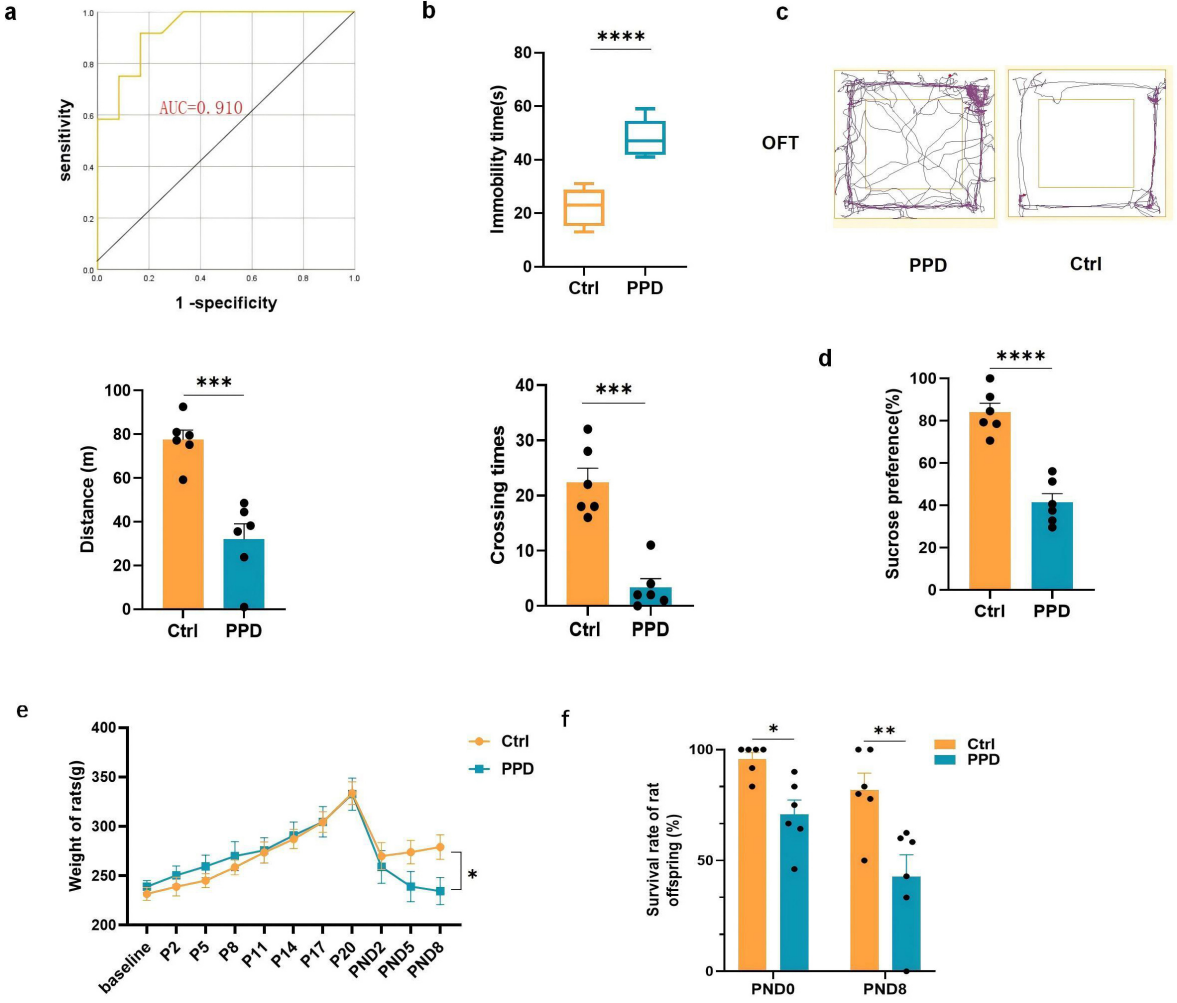
**Depression-like behavior tests in both groups**. (a) Receiver operating characteristic (ROC) curve analysis of the immobility time in both groups (n = 12). (b) Forced swim test (FST) on 5th postnatal day (PND5): the immobility time in both groups (n = 6). (c) Results of the open field test (OFT) on PND6: the total distance traveled by the rats (n = 6) and the number of line crossings (n = 6). (d) Sucrose preference test (SPT): the percentage of sucrose intake volume divided by the total fluid intake volume in both groups on PND8 (n = 6). (e) Weight changes from baseline to PND8 (n = 6). (f) Survival rate of offspring on PND0 and PND8. *p < 0.05, **p < 0.01, ***p < 0.001, ****p < 0.0001. AUC, area under the ROC curve; PPD, postpartum depression; PND, production day.

Depression-like behavior was assessed via the forced swim test at postnatal Days 4 (15 min pretest session) and 5 (6 min test session) of treatment. The latency and duration of immobility and activity (climbing and swimming) were automatically analyzed via video tracking software (Videotrack3.3, VIEWPOINT, Lyon, France). We collected immobility time data from both groups and plotted receiver operating characteristic (ROC) curves. The optimal cutoff value was 34 s, corresponding to a sensitivity of 0.833 and specificity of 0.833. The area under the ROC curve (AUC) was 0.910 (95% confidence interval (CI): 0.797–1.000, *p *= 0.01) (Fig. 1a). Therefore, an immobility time of >34 s was defined as a depressive state. Two rats exhibiting depressive symptoms in the control group and two rats without postpartum depression in the PPD group were excluded. Four rats from each group were excluded because CSF could not be collected at all time points. Therefore, six rats in the control group and six in the PPD groups were included in the final study.

The immobility times of the enrolled rats were reduced in the Ctrl group than in the PPD group (Fig. 1b). There were significant differences between the two groups (*p* < 0.0001).

Depressive-like behavior was also assessed via the OFT on postnatal Day 6 (PND6) (Fig. 1c). Compared with the mothers in the control group, the mothers in the PPD group exhibited a shorter movement distance (*p* < 0.001) and fewer crossings in the OFT (*p *= 0.0001). Additionally, on PND8, mothers with PPD experienced significant decreased interest in sucrose intake (*p* < 0.0001) (Fig. 1d) and weight loss (*p* = 0.037) (Fig. 1e). Maternal behaviors, particularly arched-back nursing, were reduced in the PPD group. This reduction even affected the survival rate of the offspring on PND0 and PND8 (PND0: *p*1 = 0.043; PND8: *p*2 = 0.002) (Fig. 1f).

### Reduced Oxytocin Levels in the PVN and CSF due to Chronic Stress During Pregnancy

**Fig. 2.**
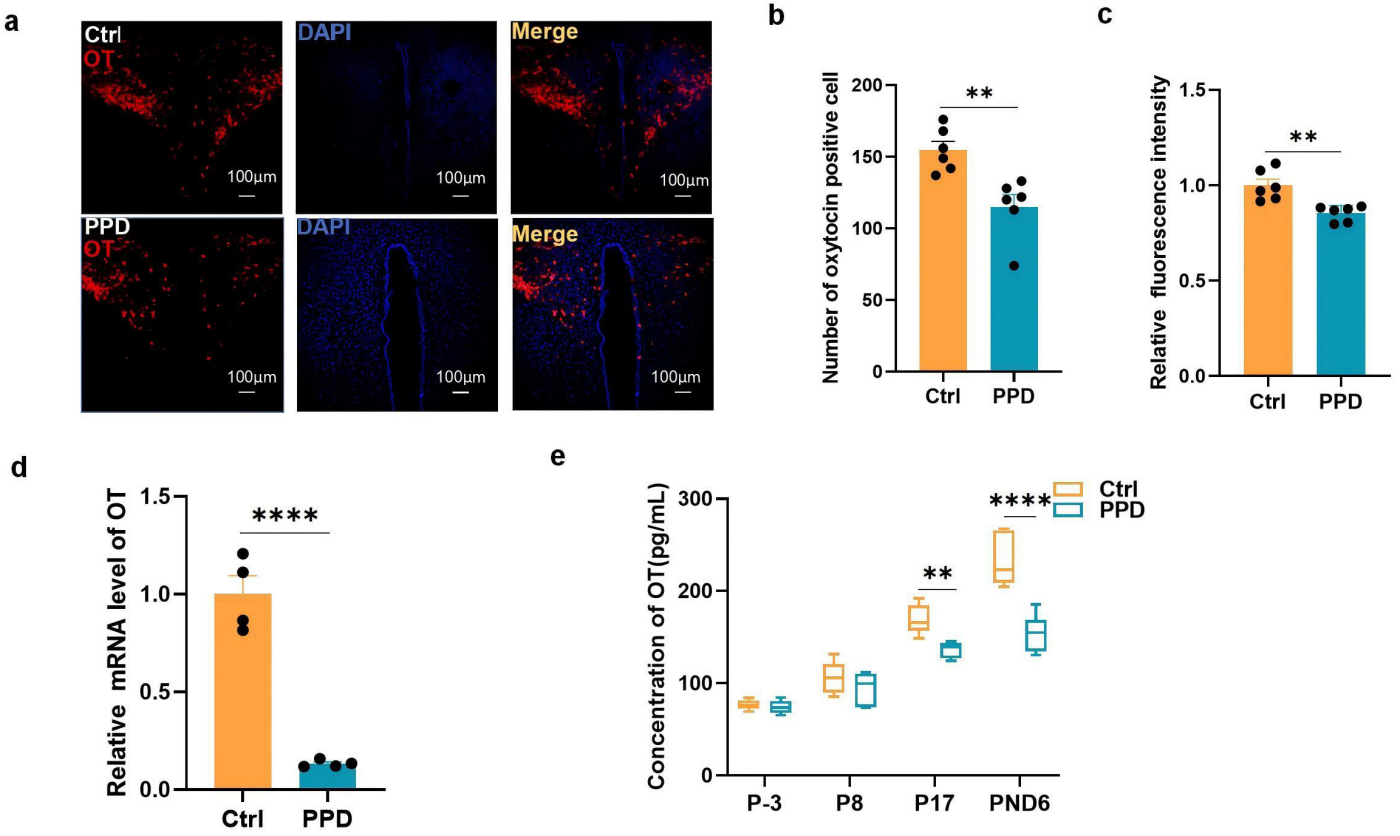
**Changes in OT levels in the brain and cerebrospinal fluid (CSF)**. (a) OT levels in the paraventricular nucleus (PVN) on PND8 (scale bar = 100 µm). (b) The number of OT-positive cells was determined via immunofluorescence (IF) staining (mean ± standard deviation (SD), n = 2 × 3). (c) IF intensity of the OT-positive cells (mean ± SD, n = 2 × 3). (d) OT mRNA expression levels were determined by qRT-PCR on PND10 (mean ± SD, n = 4). (e) OT levels in the CSF of rats were determined via ELISA throughout the perinatal period (mean ± SD, n = 6). **p < 0.01, ****p < 0.0001. ELISA, enzyme-linked immunosorbent assay; DAPI, 4^′^,6-Diamidino-2-phenylindole; OT, oxytocin.

To examine the relationship between the OT system and PPD, we collected frozen brain tissue from rats on PND8, and IF staining was used to label the OT-secreting neurons in the PVN (Fig. 2a). We counted the number and IF intensity of the OT-positive cells, which were significantly lower in the PPD group than in the control group (*p *_number_ = 0.0039; *p *_IF_ = 0.0025; Fig. 2b,c). Furthermore, qRT-PCR was conducted to measure the mRNA expression level of OT in the PVN of the hypothalamus, revealing a nearly five-fold reduction in OT expression in the PPD group compared with that in the control group (*p* < 0.0001) (Fig. 2d).

In addition, we monitored dynamic changes in the CSF OT concentration throughout the perinatal period. The ELISA results revealed that OT levels continuously increased throughout pregnancy in normal female rats. This trend was disrupted by chronic stress during pregnancy, leading to a decrease in OT levels from postconception until the postpartum stage in the PPD group compared to those in the normal group (Fig. 2e). There were significant differences between the two groups (P17: *p* = 0.0056; PND6: *p* < 0.0001), which might contribute to PPD behavior.

### Chemogenetic Excitation of Oxytocin Neurons Evokes OT Release and Improves PPD-Like Behavior

**Fig. 3.**
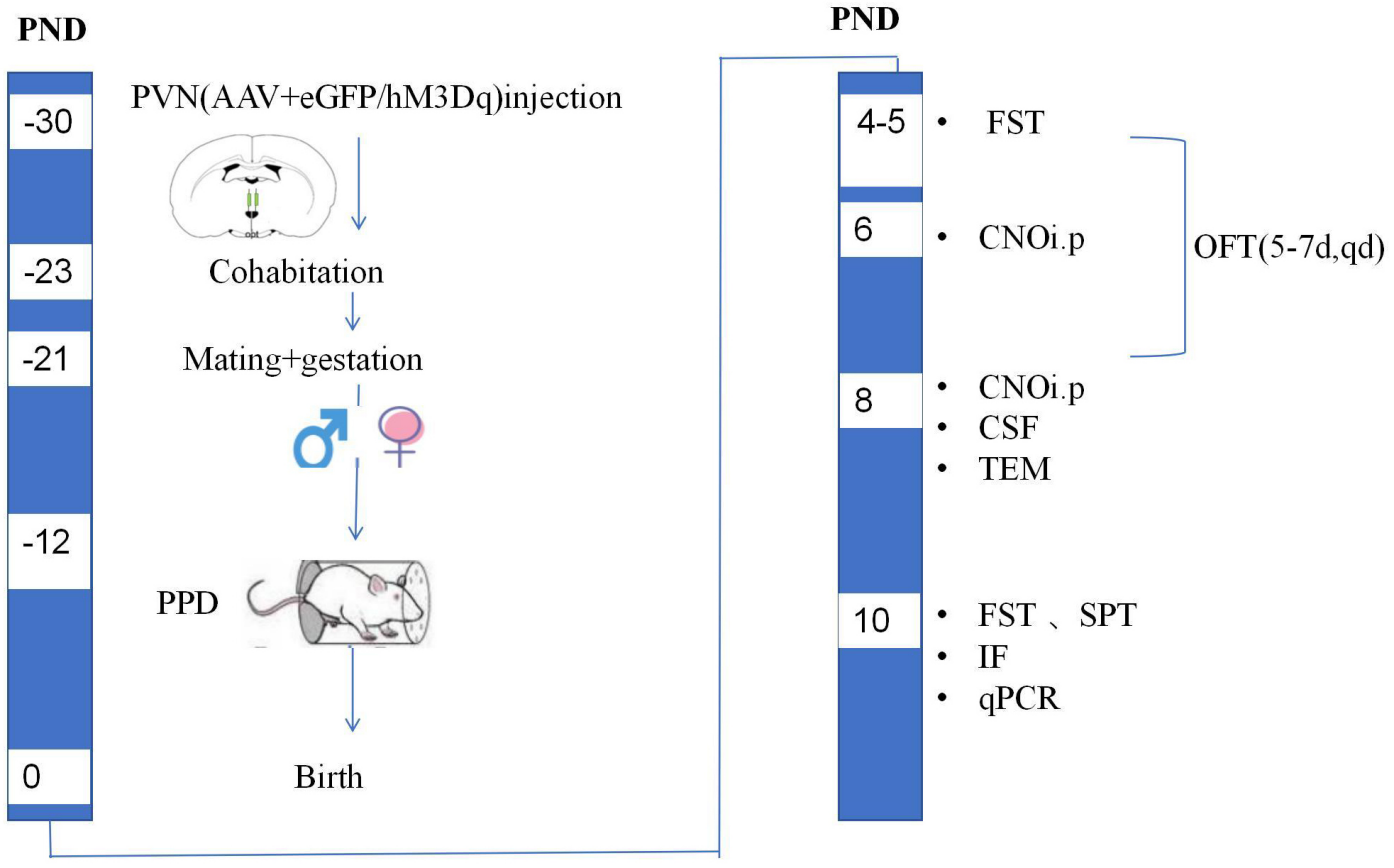
**Experimental timeline of the second stage of the experiment**. CNO, clozapine-N-oxide; TEM, transmission electron microscopy; IF, immunofluorescence; qPCR, quantitative real-time PCR.

The first part of the study revealed that lower OT levels in the PVN are associated with the occurrence of PPD-like behavior in rats. To confirm the importance of OT-secreting neurons in PPD modulation, we administered the chemogenetic virus hM3Dq to the PVN and intraperitoneally injected CNO to activate endogenous OT release. We investigated the changes in OT-secreting neurons at the cellular and molecular levels and the effects of endogenous OT on rat behavior. The experimental procedure is illustrated in Fig. 3.

**Fig. 4.**
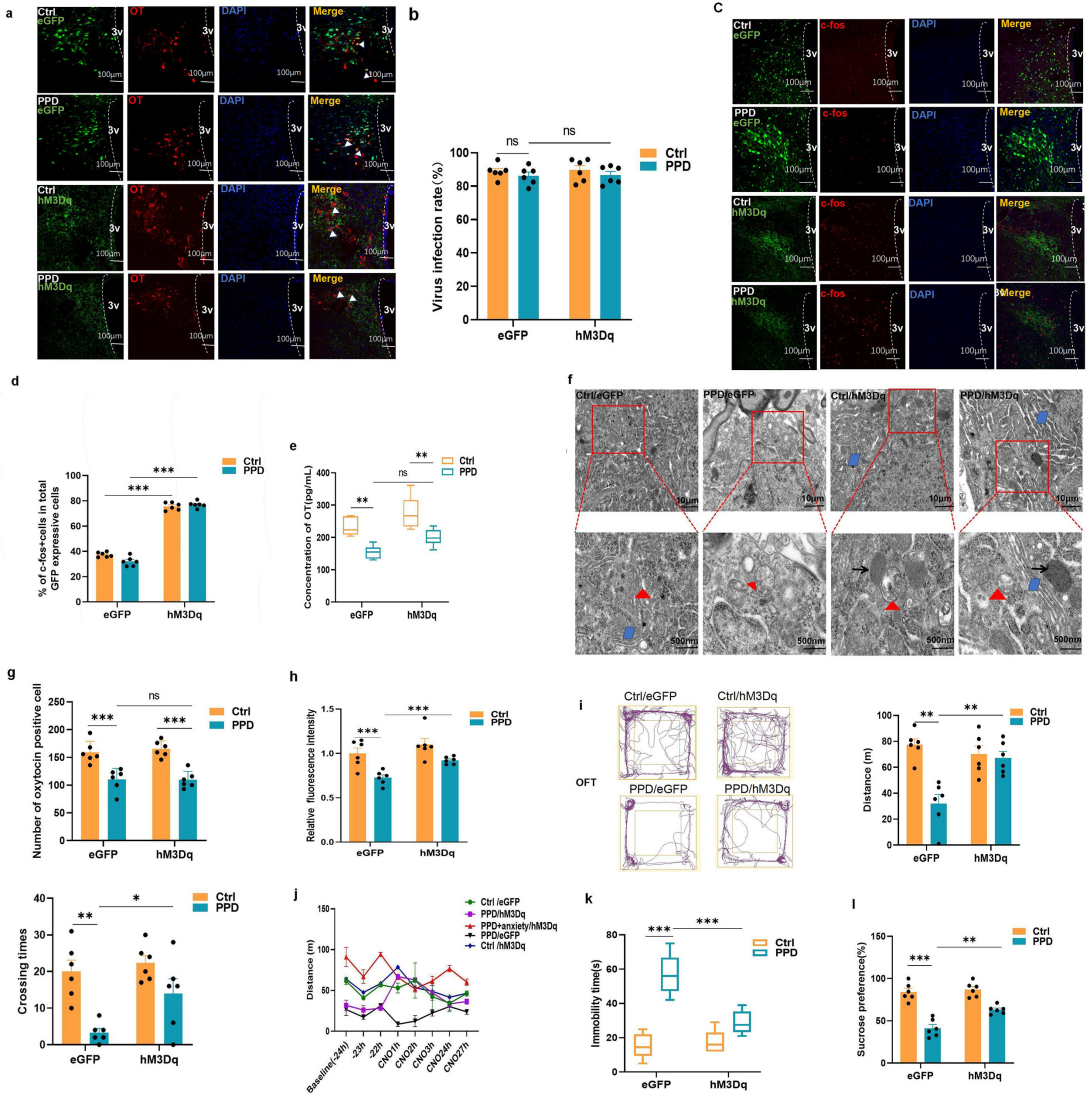
**Activation of OT-secreting neurons (hM3Dq expression) promotes endogenous OT release and improves PPD-like behavior in rats**. (a) hM3Dq and OT colocalized in the PVN (scale bar = 100 µm). (b) Viral infection rate of OT cells in the PVN (mean ± SD, n = 2 × 3). (c) Chemogenetically activated c-fos expression in OT-secreting neurons: colocalization of hM3Dq and c-fos in OT-secreting neurons in the PVN (scale bar = 100 µm). (d) Proportion of virus-expressing c-fos-expressing cells (mean ± SD, n = 2 × 3). (e) The OT concentration in the CSF of the rats was monitored by ELISA on PND8 (mean ± SD, n = 6). (f) Electron microscopy observations of OT neurons after clozapine-N-oxide (CNO) treatment for 2 h on PND8 (n = 2, →: secretory vesicles; △: large dense nuclei; ⋄: endoplasmic reticulum and Golgi apparatus; scale bar = 10 µm; scale bar = 500 nm). (g) The number of OT^+^ cells increased after chemogenetic activation (mean ± SD, n = 2 × 3). (h) IF intensity of OT^+^*/hM3Dq*^+^ cells (mean ± SD, n = 2 × 3). (i) Results of the OFT on PND6: the total distance traveled by the rats (mean ± SD, n = 6) and the crossing times after CNO treatment for 1 h (mean ± SD, n= 6). (j) OFT results on PND5-7. (k) Immobility time in the FST on PND10 (mean ± SD, n = 6). (l) SPT: the percentage of sucrose intake divided by total fluid intake on PND10 (mean ± SD, n = 6). ns, no significance; *p < 0.01, **p < 0.01, ***p < 0.001.

On PND10, 1 h after the final dose of CNO, viral expression in the OT-secreting neurons of the PVN was confirmed (Fig. 4a). We calculated the number of costained cells (OT^+^ and virus)/the number of OT^+^ cells and detected efficient and highly specific viral infection, and there were no significant differences among the four groups (F = 0.542, *p* = 0.659) (Fig. 4b).

Furthermore, IF staining revealed that treatment with CNO elicited robust c-fos expression in the PVN OT-secreting neurons of the hM3Dq AAV-injected rats compared with those in the control virus-injected rats (Fig. 4c). The efficiency of OT neuron activation was assessed by the number of costained cells (*hM3Dq*^+^ and c-fos^+^)/number of OT^+^ cells. Compared with that in the *eGFp *^+^-expressing rats, the percentage of c-fos positive cells was greater in the *hM3Dq*^+^-expressing rats (PPD/eGFP: vs. PPD/hM3Dq: *p* < 0.001; Ctrl/eGFP: vs. Ctrl/hM3Dq: *p* < 0.001; Fig. 4d), which implies that *hM3Dq*-expressing OT cells were efficiently activated and manipulated to facilitate the release of endogenous OT.

The OT concentration in the CSF of the rats was determined via ELISA 2 h after CNO injection on PND8. The ELISA results revealed lower OT levels in the PPD group than in the control group (*p* < 0.01). Compared with those in the PPD/eGFP group, the OT levels in the PPD/hM3Dq group did not increase in response to hM3Dq-induced OT release (Fig. 4e), which might be related to the timing of CSF collection.

At the light microscopy level, we observed a decrease in the abundance of the endoplasmic reticulum, Golgi apparatus, secretory vesicles, and large dense nuclei in the OT-secreting neurons in the PPD/eGFP group compared with those in the Ctrl/eGFP group. On PND8, extensive proliferation of the endoplasmic reticulum and Golgi apparatus was evident in the *hM3Dq*^+^ OT-secreting neurons. Additionally, there was an increase in the number of vesicles on neuronal cell membranes and dendrites, indicating that chemogenetic activation not only significantly enhanced the release of OT but also promoted intracellular synthesis (Fig. 4f).

OT-secreting cells synthesize OT and produce the neurotransmitters gamma-aminobutyric acid (GABA) and glutamine. To identify OT levels, we used IF technology to semiquantitatively assess OT levels in the PVN region. The results revealed the OT^+^/*hM3Dq*^+^ cell counts did not obviously increase (*p* > 0.999, Fig. 4g); however, an increased intensity of OT IF in the PPD/hM3Dq group compared with that in the PPD/eGFP group (*p* < 0.001, Fig. 4h).

Next, we investigated whether the activation of OT release could alleviate PPD behavior in maternal rats. To minimize the stress caused by the FST in female rats, we initially conducted repeated OFT assessments on PND5–7. We identified the time point at which CNO was injected at 0 h on PND6. One hour after CNO injection, there was a significant increase in the movement distance (*p* = 0.0018) and crossing time in the hM3Dq^+^-expressing group (*p* = 0.044) compared with those in the PPD group (Fig. 4i). In addition, we recorded the movement distance of the rats in the baseline period (–24 h, –23 h, and –22 h), the phase of activation (1 h, 2 h after CNO injection), and the recovery period (3 h, 24 h, 27 h). Within the PPD group, three rats displayed depressive-anxiety behavioral patterns distinct from those of the other depressed rats. During the baseline period, the rats with depressive–anxiety behavior presented a longer baseline movement distance (ranging from 45–120 m) than did the rats in the depressive state (15–45 m) and an even longer movement distance than did the rats in the control group (30–80 m). During activation period and recovery period, rats with depressive-anxiety symptoms showed the opposite behavior after CNO injection and tended to exhibit reduced horizontal movements (50–80 m), indicating that activated OT release may play antidepressant and antianxiety roles (Fig. 4j).

Finally, depressive-like behavior was assessed via the FST and SPT on PND10. Compared with that in the PPD/eGFP group, the immobility time was significantly lower in the PPD/hM3Dq group (*p* < 0.001, Fig. 4k), and the percentage of sucrose intake was increased (*p *= 0.0021, Fig. 4l).

## Discussion

The present study revealed that chronic restraint stress successfully induced a PPD rat model associated with decreased OT levels in the CSF and PVN. CNO-hM3Dq-induced activation of OT neurons in the PVN reversed lower brain OT levels and depressive-like behavior.

### Chronic Restraint Stress-Induced Depressive-Like Behaviors in the OFT, FST, and SPT

It is well known that the FST, SPT, and OFT are classical tests used to assess depressive behaviors. Previous literature has reported that chronic stress prior to pregnancy potentiates long-lasting postpartum depressive-like behavior in mice [27]. Our results also showed that chronic restraint stress during pregnancy successfully induced a depressive-like behavioral phenotype in the FST. However, two rats in the control group exhibited depressive symptoms, which has not been reported in the previous studies. This suggests that the natural fluctuations in hormones during pregnancy and the puerperal period are also causes of PPD and that the mechanism of PPD is multifactorial and complex.

In addition, we found that an immobility time of >34 s is valuable for differentiating a depressive state. The value is much lower than those previously reported [28, 29], which may be related to the definition of depression in rats (suspended in water without any limb movement) and the severity of depression.

### Chronic Restraint Stress-Induced a Decrease in OT Expression in the PVN and CSF of PPD Rats

Most OT is synthesized and released in OT neurons in the PVN. It is a small peptide neurohormone with a molecular weight of 1000 kDa that passes through the intercellular space of ependymal cells freely but through the junctions of the blood‒brain barrier (BBB) with difficulty. Therefore, OT level changes in the CSF may more accurately reflect the metabolism of OT in the brain than the levels in the plasma, which was confirmed in our previous research [13]. In this study, we monitored the OT concentration in the CSF throughout the perinatal period. The results indicated that when rats were continuously exposed to chronic restraint stress, the increase in OT levels decreased from late pregnancy to postpartum lactation. In human, these temporal dynamics of OT levels throughout pregnancy also predict postnatal mother behavior [30]. Therefore, we conclude that OT levels in the brain are negatively correlated with PPD, and the decrease in OT levels may be attributed to impaired release mechanisms within the OT system.

### Activated OT Neurons in the PVN Promote Endogenous OT Release

We used chemogenetic viral techniques to manipulate the release of OT-secreting neurons. This activated release process can occur multiple times and respond to OT changes in the CSF. However, IF staining revealed that hM3Dq increased the OT intensity, whereas the CSF OT levels did not significantly change, which might be related to the source and collection time of the OT samples. One hour after CNO injection, frozen sections of rat brain tissue were obtained, and the IF staining results indicated OT synthetic enhancement in the PVN. OT levels in the CSF do not increase because OT release peaks at 1 h after CNO injection and then gradually decreases, and the effects almost disappear after two hours, this speculation comes from the behavioral performance of rats in the OFT. CSF samples were collected at 2 h after CNO injection, which may have contributed to the negative results.

C-fos is a transcription factor that is typically expressed in the nucleus and is a marker of activity in the neuroendocrine system [31]. Research has shown that some c-fos expression occurs outside of the PVN, which may be linked to virus leakage or the activation of downstream neural pathways in OT neurons, suggesting that activated OT release in the PVN can further activate other neurons in the brain and has complex biological effects.

### Activated OT Release Might Promote OT Synthesis and Improve PPD-Like Behavior in Rats

Previous studies have focused on the regulation of emotion, cognition and behavior by OT neural circuit mechanism. Our study is the first to propose that activated oxytocin release may ameliorate postpartum depressive behavior through increased synthesis of oxytocin in the brain.

In electron microscopy images, we observed a significant increase in the number of endoplasmic reticulum, Golgi apparatus and dense nuclei following OT release, indicating the activation of OT synthesis. However, more evidence is needed to prove this synthetic effects.

The release of OT is regulated by different calcium channel mechanisms. OT can be released slowly from both the axonal and somato-dendritic compartments, diffusing in the extra-synaptic space and acting as a long-lasting neuromodulator. Somatodendritic OT release can activate transient receptor vanillin type 2 calcium channel (TRPV2) in the somata of OT neurons, leading to an increase in intracellular Ca2^+^ levels and priming OT-containing vesicles for immediate release in dendrites [32, 33]. Therefore, the levels of OT-OTR in somatodendritic compartments play a role in regulating the release of OT and contribute to positive feedback regulation within the OT-OTR-OT system.

Activated OT release improves PPD-like behavior in rats. Unlike previous experiments, we employed repeated OFT trials for the first time to monitor the ameliorative effects of depression on rat behavior. The experimental findings demonstrated that activation and release of oxytocin (OT) could induce a transient improvement in the behavior of depressed rats. However, during the recovery period (24 h–27 h), normal rats exhibited reduced horizontal movement distance compared to their baseline levels. This suggests that OT activation and release may deplete its brain storage and influence its release level under normal rhythms, leading to a delayed restoration of their typical behavioral rhythm. Since synthesis of OT requires time, intermittent manipulation of OT release is necessary.

Moreover, activated OT release produces different behavioral effects in rats with PPD. In the open-field experiment, after activating OT neuron, three anxious and depressed rats showed better and more sustained behavioral improvements compared to the depressive rats. Severely depressed rats even exhibited anxiety, head-banging, and suicidal behaviors. These behavioral differentiations indicate that the effectiveness of OT treatment for anti-anxiety or anti-depression depends on the specific mental state of rats.

The role of the OT system in the regulation of PPD is complex. Recently, a new fluorescent sensor has been reported for detecting oxytocin signals, revealing periodic increases in OT levels every 2 hours in mice through central release, termed “OT oscillation” [34]. This rhythmic release mechanism may partially explain the differential effects of OT in the treatment of postpartum depression. Therefore, our study on the mechanism of OT production helps to understand the dynamic balance between OT synthesis and release, which may be beneficial for the treatment of PPD.

In the future, preclinical studies are needed to explore the exact dosage and frequency of OT supplementation by understanding the fluctuation interval of OT release in the brain and to establish a new series of behavioral systems to evaluate OT efficacy, such as whether OT supplementation can reconstruct the behavioral rhythms of rats. These findings will be beneficial for promoting the clinical application of exogenous OT.

This study has several limitations. In this study, the rats were injected with CNO twice (on PND6 and PND8), and the time of hM3Dq-induced OT release was too short to maintain OT synthesis and regeneration. Long-term, chronic chemogenetic stimulation may prove that OT release is beneficial for promoting OT synthesis.

## Conclusion

The present study revealed that lower OT levels in the CSF are strongly associated with the occurrence of PPD, and the release of activated OT has been shown to improve PPD-like behaviors in rats and promote intracellular OT synthesis.

## Availability of Data and Materials

The authors confirm that the data and materials supporting the findings of this study are available from the corresponding author upon reasonable request.
